# Managing Agitation Associated with Schizophrenia and Bipolar Disorder in the Emergency Setting

**DOI:** 10.5811/westjem.2015.12.28763

**Published:** 2016-03-02

**Authors:** Scott L. Zeller, Leslie Citrome

**Affiliations:** * Alameda Health System, Department of Psychiatric Emergency Services, Oakland, California; † University of California-Riverside, Department of Psychiatry, Riverside, California; ‡ New York Medical College, Department of Psychiatry and Behavioral Sciences, Valhalla, New York

## Abstract

**Introduction:**

Patient agitation represents a significant challenge in the emergency department (ED), a setting in which medical staff are working under pressure dealing with a diverse range of medical emergencies. The potential for escalation into aggressive behavior, putting patients, staff, and others at risk, makes it imperative to address agitated behavior rapidly and efficiently. Time constraints and limited access to specialist psychiatric support have in the past led to the strategy of “restrain and sedate,” which was believed to represent the optimal approach; however, it is increasingly recognized that more patient-centered approaches result in improved outcomes. The objective of this review is to raise awareness of best practices for the management of agitation in the ED and to consider the role of new pharmacologic interventions in this setting.

**Discussion:**

The Best practices in Evaluation and Treatment of Agitation (BETA) guidelines address the complete management of agitation, including triage, diagnosis, interpersonal calming skills, and medicine choices. Since their publication in 2012, there have been further developments in pharmacologic approaches for dealing with agitation, including both new agents and new modes of delivery, which increase the options available for both patients and physicians. Newer modes of delivery that could be useful in rapidly managing agitation include inhaled, buccal/sublingual and intranasal formulations. To date, the only formulation administered via a non-intramuscular route with a specific indication for agitation associated with bipolar or schizophrenia is inhaled loxapine. Non-invasive formulations, although requiring cooperation from patients, have the potential to improve overall patient experience, thereby improving future cooperation between patients and healthcare providers.

**Conclusion:**

Management of agitation in the ED should encompass a patient-centered approach, incorporating non-pharmacologic approaches if feasible. Where pharmacologic intervention is necessary, a cooperative approach using non-invasive medications should be employed where possible.

## INTRODUCTION

Individuals with bipolar disorder or schizophrenia are vulnerable to episodes of agitation, which can be defined as excessive verbal and motor behavior, especially during exacerbations of their disease.^1^ Agitation associated with psychosis is a frequent reason for emergency department (ED) visits by patients with psychiatric disorders, and requires immediate action to prevent escalation to a level that could put patients, staff, and others at risk.^1^ As specialist psychiatric support other than social work services is often not available in the emergency setting, agitated patients may often need to be medically evaluated and treated by emergency physicians. The physician should, where possible, identify the underlying etiology of the agitation – whether due to an underlying non-psychiatric medical condition or primarily due to a mental disorder – before deciding on an appropriate course of action and possible pharmacologic intervention.^2^

In the past, standard practice for intervening with an agitated patient frequently involved restraint and seclusion; however, this approach is associated with many negative outcomes.^3^ From the patient’s perspective, the approach does not recognize that many affected individuals are frightened, fragile, and vulnerable, with a history of traumatic experiences; while for others, their presentation in the ED may be their first experience in mental healthcare systems. A negative experience at this stage can potentially influence their future cooperation with healthcare workers and jeopardize future management of a potentially serious underlying condition. For the medical profession, the restraint and seclusion approach, although perceived by many to be efficient, is resource intensive as there is a requirement for one-to-one observation of a restrained or sedated patient. In addition, it is often associated with staff injuries, and it increases the length of time that individuals remain in the ED, compounding problems of overcrowding and boarding.^3^,^4^ The process of the “takedown” to place an individual in restraints may take a substantial amount of time, during which staff are at high risk of assaults and injuries. Furthermore, sedation can mask an underlying condition, thereby hindering accurate diagnosis.^2^

Guidelines are available to direct clinicians in all aspects of agitation management from triage through to pharmacologic choices. When pharmacologic intervention is deemed necessary, an array of therapeutic options administered via different routes now exists, providing both the patient and physician with treatment alternatives. The aim of this narrative review is to raise awareness of best practices for the management of agitation in the ED, and to consider the role of new pharmacologic interventions for patients with agitation associated with bipolar disorder or schizophrenia. It is recognized that physicians working in the ED must also deal with agitation occurring in association with dementia, delirium, and drug abuse, however, these areas are beyond the scope of this review.

## METHODS

The content of this narrative review was based on information contained within the Best practices in Evaluation and Treatment of Agitation (BETA) guidelines with the addition of data on new pharmacologic interventions that were identified through literature searches of PubMed using combinations of the search terms “agitation,” “bipolar,” “schizophrenia,” “emergency care,” and “emergency department.” Articles were then hand searched. Additional data included in the review are based on product prescribing information.

## DISCUSSION

### Guideline Overview

Various guidelines exist for the management of agitation,^5^ some of which provide direction for agitation associated with a particular disorder, such as bipolar disorder,^6^ or occurring in a particular setting, such as the intensive care unit.^7^ In 2012, the Project BETA guidelines were published by the American Association for Emergency Psychiatry,^1^ providing detailed guidance on various aspects of patient management including medical evaluation and triage, psychiatric assessment, verbal de-escalation of the agitated patient, psychopharmacologic approaches, and the use and avoidance of seclusion and restraint.^2^,^3^,^8^–^10^ In addition, the Centers for Medicaid Services Conditions of Participation for Hospitals include mandatory regulations on the use of seclusion and restraint.

### Medical Evaluation and Triage

Agitation can be caused by disparate medical and psychiatric conditions including head trauma, infection, thyroid disease, substance abuse/withdrawal, psychotic disorders, and depression.^10^ Identifying the etiology therefore represents a significant challenge, which is made more difficult by the immediate need to calm the patient to avoid escalation.

Rating scales have been developed to measure agitation, including the single-item Behavioral Activity Rating Scale (BARS), the five-item Positive and Negative Syndrome Scale (PANSS) Excited Component (EC), and the more complex Overt Agitation Severity Scale.^11^–^18^ PANSS-EC and BARS have been successfully used as primary outcome measures in the commercial development of several agents for the indication of agitation associated with schizophrenia and/or bipolar mania. BARS is simple to use and does not require the participant/patient to answer questions, so it is favored for purely pragmatic purposes and is also useful in a non-medical setting.

For agitated patients presenting in the ED, medical evaluation and triage should include a brief history and vital signs.^10^ Where possible, oxygen levels and blood glucose levels should also be obtained. Patients with loss of memory or disorientation, severe headache, extreme muscle stiffness or weakness, heat intolerance, unintentional weight loss, new-onset psychosis, or difficulty in breathing should be immediately evaluated by a clinician.^10^ Abnormal vital signs, overt trauma, slurred speech, unequally dilated pupils, lack of coordination, seizures, or hemiparesis also warrant immediate evaluation.^10^

If feasible, attempts at de-escalation should be made at this stage in order to gain the patient’s cooperation and participation in the evaluation. There may, however, be instances where patients require medication during the assessment to calm them enough to allow a thorough evaluation. Some patients may require medication, restraint, and increased behavioral support if the risk of violent behavior becomes high and a patient remains uncooperative.^10^

Determining whether there is a known psychiatric illness is an important aspect of triage and initial evaluation, as an underlying condition would influence subsequent treatment decisions. Agitation arising from a general medical condition should be suspected for cases of new-onset agitation and for patients with a concerning past medical history, or if the onset is outside the normal ranges of psychiatric disease. A workup for a general medical condition should aim to identify the most likely underlying causes.^10^

### Psychiatric Assessment

Severe agitation can preclude the ability for emergency physicians to conduct a complete psychiatric evaluation at the outset; however, a brief evaluation should be conducted to establish the most likely cause of the agitation.^9^ In many cases, the initial assessment can be conducted through visual observation of the patient during attempts at de-escalation, combined with verbal reports from other team and family members.^9^ Next, attempts should be made to establish if the patient has delirium, other cognitive impairment, intoxication or withdrawal, a known psychiatric condition, or another cause. When the patient is calm enough – either as a result of verbal de-escalation or initial medication – a formal psychiatric evaluation should be conducted.^9^ Of note, the goal of an emergency psychiatric assessment is not necessarily to obtain a definitive diagnosis, but instead it should aim to establish a reasonable differential diagnosis, identify issues related to safety of the patient and others, and develop a suitable treatment and disposition plan.^9^

### Non-Pharmacologic Management

An important underlying principle of the Project BETA guidelines is that seclusion and restraint should be avoided, as this approach is associated with many negative outcomes.^3^,^8^ For patients and staff, injuries – both physical and psychological – often occur during restraint, which can have negative consequences that extend beyond the period during which the patient is restrained. Furthermore, restraint can damage short- and long-term patient–physician relationships.

Restraining patients can also result in additional resource use and a longer time spent in the ED. For example, in a prospective evaluation of over 1,000 adults treated in the ED, use of restraint resulted in patients spending an additional 4.2 hours in the ED compared with those not requiring restraint.^4^ Reduced ED boarding can increase hospital revenue if bed capacity is effectively managed.^19^ The need for additional staff for the restraint procedure and subsequent observation is time consuming, costly, and stops staff from performing other duties. Patients who have been sedated also spend longer in the ED, as it can be more challenging to admit or transfer a recently restrained patient or one who has been sedated.

Instead of restraint, where possible, initial attempts to calm the patient should focus on non-coercive approaches involving verbal engagement, establishment of collaborative relationship, and verbal de-escalation (Table 1).^3^ Key aspects of de-escalation include: respecting a patient’s personal space; avoiding provocation; establishing verbal contact and providing orientation and reassurance; communicating simply and concisely; identifying the patient’s wants and feelings; listening to what the patient is saying; setting clear limits; offering choices and optimism; and debriefing the patient if involuntary intervention has been necessary.^3^ As part of this strategy, non-verbal interventions, e.g. voluntary medication and environment planning, can also be useful. As discussed later, in situations where medication is taken voluntarily, some of the newer modes of administration – inhalation and rapid-onset oral medications – may be more acceptable to patients than traditional injectable formulations.

Implementation of non-coercive approaches may require changes in organizational culture and staff training;^8^,^20^ however, the benefits are widespread, including reduced resource use, costs, and staff and patient injuries, and better patient–physician relationships.^4^,^19^ The advantages and disadvantages of non-pharmacologic approaches are outlined in Table 2.

### Pharmacologic Management

Management of agitation is multifaceted and pharmacologic interventions represent only one part of the overall approach. In some cases, agitation can be managed through non-pharmacologic approaches, such as verbal interventions and de-escalation; however, for many individuals some pharmacologic treatment will be necessary.^2^ When choosing the optimal treatment, the provisional diagnosis should be taken into account (intoxication, psychiatric illness, delirium, head trauma, infection, etc.) and where possible the underlying etiology should be targeted. Consideration should also be given to the timing and extent of medication. Elderly patients pose special challenges in terms of potential comorbidities and potential drug–drug interactions, necessitating dosage adjustments.

Early and excessively aggressive pharmacologic intervention can mask underlying conditions, delaying and impeding accurate diagnosis.^2^ However, delays in medication use can allow the agitation to escalate, putting the patient, staff, and others at increased risk of harm. Furthermore, if the agitation becomes markedly more pronounced, higher doses and more frequent administration of medication may become necessary. Taking these factors into account, the goal of pharmacologic intervention should be to calm the patient to allow assessment, avoiding sleep if possible. Sleeping or over-sedated patients can require additional monitoring, which increases the burden on available resources (such as the need for one-to-one observation, assistance in toileting, etc.), and can delay appropriate disposition. The Project BETA guidelines recommend that patients should be involved in the process of selecting the drug type and administration route if possible. If the patient is able to cooperate with taking oral medications, these are preferred over intramuscular formulations.^2^

Medications commonly used in the management of acute agitation include first- and second-generation antipsychotics, and benzodiazepines. Not all interventions and/or formulations have received U.S. Food and Drug Administration (FDA) approval for this use, and they also vary in terms of strength of the experimental evidence supporting their use. For patients with agitation associated with a psychiatric disorder, such as bipolar disorder or schizophrenia, antipsychotics are preferred over benzodiazepines because they address the underlying psychosis.^2^ If, however, an initial dose of an antipsychotic does not control the agitation, the addition of a benzodiazepine is recommended over an increased dose of the same antipsychotic or addition of a second antipsychotic.^2^ Moreover, in the case of acute withdrawal from alcohol or benzodiazepines the preferred medication intervention is a benzodiazepine, e.g. lorazepam; this is not a trivial consideration, as it is estimated that approximately half of all patients with schizophrenia have a comorbid drug- or alcohol-abuse problem.^21^

Desirable features of antipsychotics are rapid onset, control of aggressive behavior, reliability, and preservation of the physician–patient relationship.^22^,^23^ Intramuscular injection enables direct entry of the active agent into the systemic circulation through the muscle’s vasculature, providing the potential for rapid onset of action. The first-generation injectable antipsychotic haloperidol has long been used in the treatment of agitation in schizophrenia.^2^ When delivered via intramuscular injection, peak plasma levels of haloperidol are reached in ~20 minutes (Table 3).^24^ This rapid onset of action must be balanced against haloperidol’s adverse-event burden, including lengthened electrocardiogram QTc interval, extrapyramidal symptoms, and akathisia.^23^ Dystonic reactions, including laryngospasm, oculogyric crisis, and torticollis, are particularly frightening for patients, and can occur 12–24 hours after administration.^25^ The occurrence of adverse effects such as these is an important consideration because they can complicate management and compromise future care as patients may be less willing to take medicines, particularly if they have experienced an acute dystonic reaction.

Intramuscular preparations of the second-generation antipsychotics ziprasidone,^26^ olanzapine,^27^ and aripiprazole^28^ have more favorable extrapyramidal side-effect profiles than haloperidol while providing similar effect sizes for the reduction of agitation.^2^,^29^ Intramuscular injections of these agents are approved by the FDA for treatment of acute agitation associated with schizophrenia (aripiprazole, olanzapine, and ziprasidone)^26^–^28^ and bipolar mania (olanzapine and aripiprazole)^27^,^28^ and they are now recommended over the first-generation antipsychotics in guidelines.^2^

One of the key disadvantages of intramuscular injections is that patients may resist, resulting in the need for manual immobilization, risking injury to healthcare providers, including inadvertent needlestick injuries. Furthermore, the use of force to immobilize the patient can result in mental trauma that has the potential to negatively affect immediate and future patient–physician relationships.

The disadvantages of intramuscular injections have led to the recommendation that non-invasive formulations should be used in situations where the patient is able to cooperate.^2^ Non-invasive formulations require at least some cooperation from patients but have the potential to prevent escalation and improve the experience of patients, and could be considered when negotiation is possible. Oral formulations of most first- and second-generation antipsychotics are available; however, administration results in entry to the systemic circulation via the portal system, absorption can be erratic, and onset of action is slower than for agents administered via intramuscular injection (Table 3).

Orally disintegrating formulations of olanzapine, risperidone, and aripiprazole have been developed, which dissolve with saliva in the mouth and can be swallowed without additional liquid.^27^,^28^,^30^ This can be beneficial for patients with dysphagia and also in patients who might divert the medication. However, this method of administration does not improve time to onset as the medication must still be swallowed, with absorption taking place lower in the gut.^32^ All three of these orally disintegrating antipsychotic formulations are bioequivalent to the regular oral tablets and provide similar efficacy and safety at the same doses.^27^,^28^,^33^,^34^

Another orally disintegrating tablet formulation of an atypical antipsychotic that is available is sublingual asenapine,^35^ which is approved by the FDA for the treatment of schizophrenia and for manic/mixed episodes associated with bipolar disorder. In contrast to the orally disintegrating tablets of olanzapine, risperidone, and aripiprazole, sublingual asenapine is absorbed in the oral mucosa and peak plasma concentration is reached in 30–60 minutes.^35^ Administration via this route has the advantage of avoiding first-pass metabolism; however, as with all oral medications, treatment requires patient cooperation. In a randomized, double-blind, placebo-controlled trial for acute agitation, sublingual asenapine was efficacious, with an effect size comparable to that observed in prior studies of intramuscular second-generation antipsychotics.^36^ However, sublingual asenapine is not approved by the FDA for acute agitation and its use for this indication would be considered off label.^35^

A recent addition to the armamentarium is inhaled loxapine, which is approved by the FDA for the acute treatment of agitation associated with schizophrenia or bipolar I disorder.^31^ Loxapine is a first-generation antipsychotic, which has been available for many years as an oral formulation and has an established safety and efficacy profile.^37^ It has recently been reformulated at a lower dose as an inhaled powder that can be directly administered to the lungs. This results in rapid absorption into the systemic circulation with peak plasma levels being reached within two minutes of administration.^31^ The efficacy and safety of inhaled loxapine for acute agitation were demonstrated in two Phase III clinical trials, one in schizophrenia and the second in bipolar mania.^38^,^39^ In these studies, the effect sizes were comparable to those observed in analogous studies of intramuscular injection of antipsychotics or lorazepam.^40^ Of note, clinical effects, as measured by separation from placebo on the PANSS-EC, were observed as early as 10 minutes after inhalation, the first time point that this was measured.^38^,^39^ Inhaled loxapine was generally well tolerated, with dysgeusia being the most common spontaneously reported adverse event. Extrapyramidal adverse events and akathisia were relatively rare; however, spirometry studies indicated inhaled loxapine can cause bronchospasm that has the potential to lead to respiratory distress and respiratory arrest. For this reason, inhaled loxapine is restricted to use in hospitals with access to facilities to deal with acute bronchospasm, and is only available through a restricted program under a risk-evaluation and mitigation strategy. It is worth noting that as inhaled loxapine is self-administered under medical supervision, it is unlikely to be suitable in situations where patients are actively refusing treatment.^1^ However, even a patient in restraints could conceivably use voluntarily self-administered medications, if one arm can be safely released.

Midazolam – a water-soluble, fast-acting benzodiazepine – can be administered through various routes, including intranasally. Although not FDA approved for acute agitation, there has been interest in the potential use of this formulation for this indication.^32^ Intranasal midazolam is absorbed by the nasal mucosa and avoids first-pass metabolism. In children, intranasal midazolam induced calming within 15 to 20 minutes.^41^ A caveat is that midazolam is chiefly used for sedation and has no antipsychotic effects; thus, like lorazepam, it would not ameliorate hallucinations or delusions, and will not treat the underlying psychosis that may be engendering the agitation. Although using a sedation agent alone might temporarily relieve agitation, there is the risk that upon awakening, if the psychotic symptoms still persist, agitation might quickly return.

## CONCLUSION

Agitation represents a significant challenge in the ED, a setting in which medical staff are working under extreme pressure and dealing with a diverse range of medical emergencies. The potential for agitation to escalate into aggressive behavior, putting patients, staff, and others at risk, means that it is important to address the behavior rapidly and efficiently to ensure the safety of all involved. Time constraints and limited access to specialist psychiatric support have in the past led to the somewhat draconian strategy of “restrain and sedate, ‘ which was believed to represent the optimal approach. It is increasingly recognized that more humane, patient-centered approaches result in improved short- and long-term outcomes, including fewer injuries, better therapeutic alliance, improved throughput, and reduced resource use and costs. The Project BETA guidelines address the complete management of agitation, including triage, diagnosis, interpersonal calming skills, and medicine choices. Since their publication in 2012, there have been further developments in pharmacologic approaches for dealing with agitation, including both new agents and new modes of delivery, which increase the options available to patients and physicians. Older interventions, such as intramuscular haloperidol, are – in the authors’ opinion – essentially now obsolete, because effective, yet more benign, FDA-approved injectable treatments are available instead.^42^,^43^ However, despite the availability of these injectable agents, non-invasive formulations, such as sublingual, inhaled, or intranasal agents, although requiring cooperation from patients, should be used whenever possible to improve the overall patient experience, thereby potentially improving future cooperation between patients and healthcare providers. At the present time inhaled loxapine is the only non-injectable option specifically approved by the FDA for this purpose; however, evidence is also available for sublingual asenapine and intranasal midazolam.

## Figures and Tables

**Table 1:** Behavioral interventions for different scenarios involving patient agitation.

Behavioral intervention	Patient scenario
Verbal de-escalation	Should be attempted in all patients
Quiet unlocked room	Patients in whom de-escalation alone was insufficient to reduce dangerousness enough to allow to remain in general care areas, and/or may need more time to regain control away from other patients
Locked seclusion	If patients are considered an imminent danger to others but not themselves, and cannot tolerate or remain in a quiet unlocked room
Restraint	If patients are considered an imminent danger to themselves, and cannot remain in a locked seclusion room without actively trying to injure themselves.

**Table 2:** Advantages and disadvantages of non-pharmacologic interventions for agitation.

Advantages	Disadvantages
Facilitates better short- and long-term patient–physician relationships Reduces staff and patient injuries associated with restraint and sedation Reduces resource (clinical and staff) use	May not be effective in all patients Requires some co-operation from the patient

**Table 3:** Advantages and disadvantages of different routes of administration.

Administration route	Advantages^32^	Disadvantages^32^	Examples	Time to peak plasma concentration
Intramuscular	Rapid systemic entry; patient cooperation not necessary	Invasive; can damage patient–physician relationship	Haloperidol^24^	~20 minutes
			Olanzapine^27^	15–45 minutes
			Aripiprazole^28^	1–3 hours
			Ziprasidone^26^	60 minutes
Inhaled	Less invasive than intramuscular route and can improve patient experience. Enters alveoli for rapid entry into arterial circulation	Requires patient cooperation Bronchospasm/respiratory distress	Loxapine^31^	2 minutes
Oral				
Standard tablets/capsules/solution	Less invasive than intramuscular route and can improve patient experience	Require patient cooperation; slow onset of action; enter systemic circulation via portal system resulting in potential for erratic absorption; can be diverted (“cheeking”)	Haloperidol^24^	2–6 hours
			Olanzapine^27^	5–8 hours
			Risperidone^30^	~1 hour
			Aripiprazole^28^	3–5 hours
			Ziprasidone^26^	6–8 hours
Orally disintegrating tablets	Less invasive than intramuscular route and can improve patient experience. Less potential for diversion (“cheeking”) vs standard tablets/capsules; suitable for patients with dysphagia	Slow onset of action; enter systemic circulation via portal system resulting in potential for erratic absorption	Olanzapine^27^	~6 hours
			Risperidone^30^,^33^,^34^	1–2 hours
			Aripiprazole^28^	3–5 hours
Buccal/sublingual	Less invasive than intramuscular route and can improve patient experience; rapid absorption; avoids first-pass metabolism	Requires patient cooperation; needs to be taken correctly so that it is not swallowed, mitigated in part by the friability of the tablet	Sublingual asenapine^35^	0.5–1.5 hours
Intranasal	Less invasive than intramuscular route and can improve patient experience; rapid absorption; avoids first-pass metabolism	Requires patient cooperation.	Intranasal midazolam^32^	10 minutes
